# Workplace Discrimination and Mental Health in Korean Baby Boomers: The Role of Work Engagement

**DOI:** 10.1093/geroni/igaf122.2349

**Published:** 2025-12-31

**Authors:** Gaeun Han, Giyeon Kim

**Affiliations:** Chung-Ang University, Seoul, Republic of Korea; Chung-Ang University, Seoul, Republic of Korea

## Abstract

As Korean Baby Boomers, born between 1955 and 1974, continue to participate in the workforce actively, they face workplace discrimination, which negatively affects their mental health. Work engagement can mitigate the negative effects of workplace discrimination but is also influenced by those experiences. Using data from the 7th Korean Working Conditions Survey (KWCS) conducted in 2023, this study investigates the mediating and moderating effects of work engagement on the relationship between workplace discrimination and mental health among wage-employed Korean Baby Boomers (N = 12,113). A moderation and mediation analysis were conducted by PROCESS macro using SPSS 28.0. Results show that: first, workplace discrimination was negatively associated with both mental health (B=-.534, p<.001) and work engagement (B=-.441, p<.001), indicating that an increase in workplace discrimination experiences have a negative impact on mental health and work engagement. Second, a significant indirect effect of work engagement was found (B=-.501, CI=-.621, -.382), while no direct effect was observed, indicating an indirect-only mediation effect on the relationship between workplace discrimination and mental health. Third, work engagement moderates the relationship between workplace discrimination and mental health, with the effect being significant only for the high work engagement group (B=-.101, p<.05). However, work engagement exacerbated the negative impact of workplace discrimination on mental health. These findings contribute to the Job Demands-Resources (JD-R) model by uncovering the complex characteristics of work engagement among aging workers. The study underscores the need for targeted interventions to reduce workplace discrimination and enhance mental health among Baby Boomers, particularly through work engagement strategies.

